# Working Conditions and Individual Differences Are Weakly Associated with Workaholism: A 2-3-Year Prospective Study of Shift-Working Nurses

**DOI:** 10.3389/fpsyg.2017.02045

**Published:** 2017-11-21

**Authors:** Cecilie S. Andreassen, Arnold B. Bakker, Bjørn Bjorvatn, Bente E. Moen, Nils Magerøy, Akihito Shimazu, Jørn Hetland, Ståle Pallesen

**Affiliations:** ^1^Department of Clinical Psychology, University of Bergen, Bergen, Norway; ^2^Center of Excellence for Positive Organizational Psychology, Erasmus University Rotterdam, Rotterdam, Netherlands; ^3^Department of Global Public Health and Primary Care, University of Bergen, Bergen, Norway; ^4^Norwegian Competence Center for Sleep Disorders, Haukeland University Hospital, Bergen, Norway; ^5^Department of Occupational Medicine, Haukeland University Hospital, Bergen, Norway; ^6^Department of Mental Health, University of Tokyo, Tokyo, Japan; ^7^Department of Psychosocial Science, University of Bergen, Bergen, Norway

**Keywords:** workaholism, job demand-control-social support, individual differences, sleep, flexibility

## Abstract

This study focuses on individual differences and the demand-support-control model in relation to workaholism. We hypothesized that unfavorable working conditions (high job demands, low job control/decision latitude, and low social support at work) and individual differences concerning sleep/wake-related variables (high flexibility, high morningness, and low languidity) would be related to workaholism measured 2–3 years later. Survey data stemmed from a prospective cohort of shift-working nurses (*N* = 1,308). The results showed that social support at work was negatively related to workaholism, whereas job demands were positively related to workaholism. Flexibility in terms of time for working/sleeping was also positively related to workaholism. The analyses further revealed that workaholism was inversely associated with age as well as having a child or having a child move in. Conjointly, the independent variables explained 6.4% of the variance in workaholism, while their relative importance was small overall. After controlling for all other independent variables, high job demands had the strongest relationship (small-to-medium) with workaholism. This implies that less pressure from the external environment to work excessively hard may prevent an increase in workaholic behaviors. Overall, the study adds to our understanding of the relationships between working conditions, individual differences, and workaholism.

## Introduction

Over the past decade, workaholism has become an increasingly studied subject for empirical investigation. Workaholism is typically described as a chronic pattern of high work investment, long working hours, working beyond organizational expectations, and an uncontrollable obsession with work (Ng et al., 2007). Although the concept has been associated with positive attributes such as extraordinary work effort (Machlowitz, 1980), most scholars currently regard workaholism mainly as a negative entity (Andreassen, 2014) as it is associated with impaired health (Andreassen et al., 2007), low job and life satisfaction (Andreassen et al., 2011), work-family conflicts (Andreassen et al., 2013), sleep problems (Salanova et al., 2016), as well as impaired job performance (Shimazu et al., 2012) and sickness absence (Falco et al., 2013). In recent years, scholars have come to see workaholism more and more in terms of a behavioral addiction, defining it as “being overly concerned about work, to be driven by strong and uncontrollable work motivation, and to spend so much energy and effort into work that it impairs private relationships, spare-time activities and/or health” (Andreassen et al., 2014b, p. 8). Lack of representative studies makes the prevalence of workaholism uncertain, however, in a nationally representative survey of workers, a prevalence of 8.3% was reported (Andreassen et al., 2014a). Most workaholism studies are purely cross-sectional and relatively few predictors/correlates of workaholism have been identified so far. Arguably, there is a need to identify factors that may predict workaholism over time.

Behavior is usually the result of an interaction between individual and environmental influences, yet few scholars have conceptualized workaholism from an interactionist perspective (Mazzetti et al., 2014). This study aimed to investigate how particular working conditions (job demands, control/decision latitude, and social support) combine with a novel set of individual differences in sleep/wake variables (flexibility, languidity, and morningness) to explain variance in workaholism. A prospective design was used in which nurses are followed over the course of 2–3 years.

### The Role of the Working Conditions

One of the most cited and prevailing models in terms of organizational stressors is the demand-control-support model (Bakker and Demerouti, 2007), which consequently has been used to explain workaholism. High demands are a strain on and impair employee health, whereas control and support are assumed to work in the opposite direction, either directly (additive components), or as buffers (interactive components) (Karasek, 1979; Johnson et al., 1989). Generally, research supports the idea of direct effects on strain, but support for interactive effects is limited and inconsistent (Van der Doef and Maes, 1999; Häusser et al., 2010). Previous research has consistently shown positive associations between demands and workaholism (Johnstone and Johnston, 2005; Choi, 2013; Matsudaira et al., 2013; Shimazu et al., 2014; Molino et al., 2016). High demands typically force workers to deal with a lot of work and/or to work at high speed. This may be perceived by workers as a descriptive norm (Vallerand et al., 2003). In addition, hard workers may be perceived as role models (Kravina et al., 2014). We therefore hypothesize that job demands relate positively to workaholism (H1).

The relationship between job control and workaholism seems more ambiguous, as two previous studies found no relation (Guglielmi et al., 2012; Shimazu et al., 2012), whereas one study reported a negative relation. Employees having control in their jobs can more easily decide on their optimal pace and way of working. When autonomy is low, however, sustained activation and inability to recover may occur (Andreassen et al., 2010, 2011). Studies have suggested a negative relationship between job control and workaholism (Matsudaira et al., 2013). Since it is conceivable that some individuals may overwork in hopes of being able to handle the uncontrollable work situation, we hypothesize that job control relates negatively to workaholism (H2).

The relationship between social support from colleagues and workaholism also seems ambiguous. Two studies reported no relationship between workaholism and indicators of social support (Choi, 2013; Shimazu et al., 2014) whereas a negative relationship was found in a third study (Matsudaira et al., 2013). From a theoretical perspective, it is reasonable to assume that lack of support from colleagues facilitates workaholism, because employees may be forced to finish most of their tasks alone and/or may be motivated to work harder in order to gain respect and support at the job. Hence, we hypothesize that social support relates negatively to workaholism (H3).

### The Role of Individual Differences

The other approach to workaholism in the present study concerns individual differences, i.e., the many ways in which people differ. Personality is one key dimension to this concept and can be defined as a set of emotions, cognitions, and behaviors characterizing an individual. Emphasis has been put on the five-factor model of personality, which differentiates between five dimensions: neuroticism, extroversion, openness to experience, agreeableness, and conscientiousness (Wiggins, 1996). The most consistent finding is that neuroticism and conscientiousness associate positively with workaholism (Andreassen et al., 2010; Clark et al., 2010; Schaufeli, 2016).

However, other aspects of personality may also be important to workaholism, including stable sleep-related personality characteristics. Surprisingly little is known about the relationship between workaholism and sleep beyond studies linking workaholism to poor sleep (Kubota et al., 2014; Salanova et al., 2016). Workaholics typically work extended hours and spend much more time working than initially intended (Andreassen et al., 2012). This implies that many workaholics work outside regular working hours; hence, they are engaged in shift work. Consistent with this idea, results of additional analysis using data from the abovementioned nationally representative sample of workers showed that shift workers scored higher on workaholism than day workers (Andreassen et al., 2014a).

Shift work typically takes a toll on sleep (Åkerstedt, 2003). So far, no previous study has investigated the relationship between individual differences that relate to shift work tolerance, the ability to work shifts without experiencing negative health effects (Andlauer et al., 1979), and workaholism.

Mammals show variations in several behavioral and physiological processes such as sleep and waking times, core body temperature, work performance, and alertness (Rajaratnam and Arendt, 2001). These processes oscillate with periods of about 24 h and reflect as such circadian rhythms. These are governed by a central circadian clock (the nucleus suprachiasmaticus) as well as a clocks embodied in every cell (Mohawk et al., 2012).

Although all mammals show circadian rhythmicity, there are also individual differences related to these rhythms (Baehr et al., 2000). One relevant sleep characteristic where people differ is flexibility, which is defined as the ability to sleep and work at odd hours (Di Milia et al., 2005). People low in flexibility are assumed to have high stability of their circadian rhythms, whereas those with high scores on flexibility are assumed to have non-stable circadian rhythms which would make it easier for them to adapt to shift work (Di Milia et al., 2005). In line with this, a review paper concluded that flexibility was positively related to shift work tolerance (Saksvik et al., 2011). High ability to sleep and work at odd hours (i.e., flexibility) can thus be regarded as a prerequisite for workaholism, as it increases the ability to work around the clock and beyond what is normally expected. Thus, we hypothesize a positive association between flexibility and workaholism (H4).

Another circadian sleep characteristic that may also influence workaholic tendencies is languidity, reflecting general difficulties overcoming drowsiness and feelings of lethargy following sleep loss. This characteristic is assumed to reflect the amplitude of the circadian rhythm where high rhythm amplitude is thought to cause high levels of languidity, whereas low rhythm amplitude is assumed to cause low levels of languidity. In line with this, high rhythm amplitude/languidity is assumed to be associated with poor shift work tolerance (Di Milia et al., 2005), a notion that has been supported by previous studies (Saksvik-Lehouillier et al., 2013).

Workaholics typically are heavy work investors at all times, regardless of what kind of work they do or which organizations they work for (Harpaz and Snir, 2003). In line with this, workaholics have typically been described as persons devoting enormous amounts of time and energy to their jobs (Quinones and Griffiths, 2015). As this is incompatible with high scores on languidity, we assume that languidity relates negatively to workaholism (H5).

The most notable and best studied individual circadian characteristic is morningness, which is denoted as a preference for rising, conducting activities, and going to bed relatively early, and reflects the phase of the circadian rhythm in relation to the external environment (Thun et al., 2012). In the majority of previous studies, morningness has been found to be positively associated with shift work tolerance (Saksvik-Lehouillier et al., 2012). Previous studies have shown a link between low score on morningness and poor sleep quality (Vardar et al., 2008), sleep debt (Taillard et al., 1999) and work-related chronic fatigue, probably due to sleep incompatible behaviors (Martin et al., 2012). Hence, the potential of recovery following sleep seems lower for those scoring low on morningness, which arguably will make them less able to work in an obsessed and uncontrollable manner. Based on this, we hypothesize a positive relation between morningness and workaholism (H6).

### Interaction between Working Conditions and Individual Differences

The work context and individual differences, as noted above, do not operate independently of each other, and several studies have pointed to interactions between these sets of factors. Hence, it is conceivable that work variables and individual differences may interact in creating workaholism. Specifically, since it was hypothesized that highly demanding working conditions may potentially serve as catalysts for workaholism in disposed individuals (Choi, 2013; Matsudaira et al., 2013; Mazzetti et al., 2014; Shimazu et al., 2014; Molino et al., 2016; Schaufeli, 2016), it would be relevant to explore the interactions between job demand and flexibility, languidity, and morningness, respectively. We expect demands (which may trigger workaholism) to interact with (a) flexibility (ability to work at odd times), (b) languidity (inability to cope with little sleep), and (c) morningness (preference for morning activities) so that the effect of demand on workaholism is anticipated to be high when levels of flexibility are high, when levels of languidity are low, and when levels of morningness are high, respectively. The effects of demand on workaholism are expected to be lower when levels of flexibility are low, when levels of languidity are high and when levels of morningness are low (H7).

If working conditions, sleep, and workaholism have stable associations, then there would be substantial implications for casual models of workaholism and health. The present study is the first to examine links between workaholism with demand-control-support categories and sleep variables in terms of flexibility, languidity, and morningness in a shift-working sample of nurses, using well-validated measures.

## Materials and Methods

### Procedure and Participants

This study is based on longitudinal data from the Survey of Shift work, Sleep, and Health (SUSSH), which explores the work and health status of Norwegian nurses. The first data collection took place in 2008/2009 (T1), with annual follow-ups, whereas the fourth collection took place in 2012 (T4), a time lag of about 2–3 years.

Participants initially received a letter describing the purpose of the study and an attached letter of recommendation from the Norwegian Nurses Organization (NNO). Those who participated took part in a lottery where 50 individuals could win about $70. Informed consent was obtained in written form. Originally 6,000 nurses were invited, all members of NNO (the organization includes most nurses working in Norway, about 96,000). Nurses were randomly selected from equal strata based on years since basic nursing education (0–1 year, 1.1–3 years, 3.1–6 years, 6.1–9 years, and 9.1–12 years). A cohort of 2,059 nurses was established (38.1% response rate when removing returns due to wrong addresses) in 2008/2009; 1 year later (2009), 2,741 newly graduated nurses were invited to participate, of which 905 agreed, yielding a response rate of 33.0%. These two groups together formed the baseline cohort of the SUSSH. At T4, 2,136 participated, which amounted to 72.1% of all individuals who participated at T1.

In the present study, nurses working two or three rotating shifts at T4 were eligible (*n* = 1,308; 91.6% women). Their mean age was 31.73 years (*SD* = 8.34) at T1; 39.2% were not living with children at T1 or T4; 35.4% were living with children at T1 and T4; 19.7% were not living with children at T1 but at T4, whereas 2.5% were living with children at T1 but not at T4. In all, 28.4% reported working more than full-time equivalent at T4, and 85.2% had the same work (somatic, nursing home, etc.,) at T1 and T4. Of note, T1 was the only time where all the independent variables were measured simultaneously; whereas workaholism was first assessed at T4.

### Measures

#### Workaholism

Workaholism was assessed by the Bergen Work Addiction Scale (BWAS), which comprises seven items (symptoms) based on general addiction criteria (salience, tolerance, mood modification, relapse, withdrawal, conflict, and problems) experienced during the past year. Each item is answered on a scale from 1 (never) to 5 (always) (e.g., “become stressed if you have been prohibited from working?”). The BWAS has demonstrated a one-factor solution (single construct) and high convergent validity across studies and cultures (e.g., Andreassen et al., 2012, 2014a, 2016; Molino et al., 2016; Orosz et al., 2016). Cronbach’s alpha for the BWAS in the current study was high (0.83). In a recent critical review, it was concluded that the BWAS is adequately conceptualized within an addiction framework (Quinones and Griffiths, 2015).

#### Working Conditions

These were assessed by the Swedish Demand-Control-Support Questionnaire (DCSQ), which is a measure of the key environmental factors of the Demand-Control-Support model: *job demand* (5 items; e.g., “Does your job require too great a work effort?”), *control/decision latitude* (6 items; e.g., “Do you have the opportunity to learn new things in your work?”), and *social support at work* (6 items; e.g., “There is good collegiality at work”). Response alternatives for demand and control items are 1 (no, almost never) to 4 (yes, often); whereas alternatives for social support items are 1 (strongly disagree) to 4 (strongly agree). The DCSQ has shown good psychometric properties (Sanne et al., 2005). Current Cronbach’s alphas were 0.78 (demand), 0.49 (control), and 0.83 (support).

#### Sleep Variables

These were assessed by the revised Circadian Type Inventory (rCTI) and the Diurnal Scale (DS). The rCTI consists of 11 items concerning daily sleep, waking, activity habits, and preferences across two subscales. *Flexibility* (5 items; e.g., “Do you enjoy working at unusual times of day or night?”) refers to the ability to sleep and work at odd times, whereas *languidity* (6 items; e.g., “Do you tend to need more sleep than other people?”) is related to difficulties overcoming drowsiness and feelings of lethargy following sleep reduction. Response alternatives for flexibility and languidity items are 1 (almost never) to 5 (almost always). The rCTI has shown high reliability and validity. Current Cronbach’s alphas were 0.80 (flexibility) and 0.69 (languidity). The Diurnal Scale consists of seven items assessing the *morningness-eveningness* dimension rated on a 4-point scale to indicate preferred time for conducting certain activities (e.g., “If you always had to rise at 06:00 am, what do you think it would be like?”). The DS has shown good reliability and validity (Torsvall and Åkerstedt, 1980), and current Cronbach’s alpha was 0.64.

## Results

Descriptive and correlational (Pearson’s *r*) statistics were calculated, as shown in **Table 1**. Both statistical (*p*-values) and practical (strength of associations) significance were evaluated. Not having children at T1/T4, demand at T1, and flexibility at T1 correlated positively and significantly (*p* < 0.01) with workaholism at T4. Having children at T1/T4 (*p* < 0.05), age at T1 (*p* < 0.05), social support at T1 (*p* < 0.01), and morningness at T1 (*p* < 0.01) correlated negatively and significantly with workaholism at T4. Strengths of significant associations were relatively small (*r* = -0.07–0.17), with the strongest between demand and workaholism. Reliability of control (0.49) and morningness (0.64) were rather low.

**Table 1:** Descriptive data and correlation coefficients between study variables (*N* = 1,207–1,307).

		Mean (*SD*)/Percentage	1	2	3	4	5	6	7	8	9	10	11	12
1.	Age at T1	31.73 (8.34)
2.	Gender		-0.06
	1 = ♂	8.4%
	2 = ♀	91.6%
3.	Children at T1/T4	35.4%	0.34	0.03
4.	Children not at T1/T4	39.2%	-0.19	-0.07	-0.63
5.	Children not at T1/at T4	19.4%	-0.29	0.06	-0.37	-0.41
6.	Children at T1/not at T4	2.5%	0.26	-0.01	-0.12	-0.13	-0.08
7.	Workaholism at T4	12.86 (4.68)	-0.07	-0.03	-0.07	0.09	-0.04
8.	Demand at T1	14.31 (2.60)	-0.08	0.04	-0.00	-0.06	0.09	0.00	0.17
9.	Control at T1	17.63 (1.99)	0.03	0.07	-0.02	0.00	0.01	0.02	-0.02	-0.02
10.	Social support at T1	19.21 (2.70)	-0.05	0.03	-0.03	0.06	-0.03	-0.03	-0.10	-0.28	0.24
11.	Flexibility at T1	12.37 (3.96)	-0.08	-0.05	-0.12	0.09	0.02	-0.02	0.09	-0.12	0.03	0.09
12.	Languidity at T1	20.71 (3.61)	-0.21	0.09	-0.07	0.03	0.07	-0.05	0.04	0.15	-0.11	-0.13	-0.15
13.	Morningness at T1	17.61 (3.31)	0.04	0.08	0.16	-0.13	-0.05	0.01	-0.09	-0.02	-0.06	0.06	-0.27	-0.40

*T1, first time data (measured in 2008/2009); T4, fourth time data (measured in 2012). All correlations with an absolute value ≥ 0.06 < 0.08 are significant at *p* < 0.05. All correlations with an absolute value ≥ 0.08 are significant at *p* < 0.01.*

A hierarchical linear regression analysis was conducted to establish the associations between working conditions and dispositional sleep/wake patterns (all assessed at T1), and workaholism at T4; controlling for age, gender, and living with children. Living with children was coded as dummy; no children at both T1/T4 constituted the reference category. Results are presented in **Table 2**. Age, gender, and living with children were entered at Step 1, explaining 1.5% of the variance, with an *F*^2^ of 0.015 (very small effect). At Step 2, demand, control, social support, flexibility, languidity, and morningness were entered. These variables explained a total of 4.7% of the variance in workaholism, Δ*R*^2^ = 0.047, Δ*F*_6,1177_ = 9.78, *p* < 0.01; *F*^2^ = 0.049 (small effect). The three interaction terms between demand × sleep variables at Step 3 were all non-significant, and explained only 0.2% of the variance, Δ*R*^2^ = 0.002, Δ*F*_3,1174_ = 1.03, *p* = 0.38.

**Table 2:** Hierarchical regression analysis of the relationships between working conditions, individual differences, and workaholism.

Variables	Step 1: Demographics				Step 2: Work/Sleep				Step 3: Demand × Sleep				Model summary				
	*B*	SEB	β	*t*	*B*	SEB	β	*t*	*B*	SEB	β	*t*	*R*^2^	Δ*R*^2^	*F*_df1,df2_	Δ*F*_df1,df2_	*p*
**1. Demographics T1/T4**													*R*^2^ = 0.015				
Age T1	-0.045	0.018	-0.080	-2.453^∗^	-0.039	0.019	-0.069	-2.101^∗^	-0.037	0.019	-0.066	-1.978^∗^	*F*_5,1183_ = 3.65, *p* < 0.01				
Gender (1 = ccc, 2 = ddd)	-0.479	0.487	-0.028	-0.984	-0.393	0.482	-0.023	-0.814	-0.384	0.486	-0.023	-0.790					
Children at T1/T4^1^	-0.667	0.324	-0.068	-2.058^∗^	-0.613	0.322	-0.063	-1.901	-0.618	0.325	-0.063	-1.901					
Children not T1/at T4^1^	-1.007	0.371	-0.086	-2.710^∗∗^	-1.207	0.365	-0.103	-3.305^∗∗^	-1.199	0.368	-0.102	-3.257^∗∗^					
Children at T1/not T4^1^	0.809	0.913	0.027	0.886	0.674	0.895	0.023	0.753	0.564	0.904	0.019	0.624					
**2. Work T1/Sleep T1**													*R*^2^/Δ*R*^2^ = 0.062/0.047				
Demand T1					0.795	0.140	0.170	5.707^∗∗^	0.782	0.141	0.167	5.561^∗∗^	*F*_11,11177_ = 7.07, *p* < 0.01;				
Control T1					0.015	0.068	0.006	0.222	0.025	0.069	0.011	0.367	Δ*F*_6,1177_ = 9.78, *p* < 0.01				
Social Support T1					-0.116	0.053	-0.067	-2.208^∗^	-0.121	0.053	-0.070	-2.272^∗^					
Flexibility T1					0.394	0.146	0.084	2.725^∗∗^	0.395	0.146	0.084	2.707^∗∗^					
Languidity T1					-0.060	0.158	-0.013	-0.383	-0.060	0.158	-0.013	-0.382					
Morningness T1					-0.257	0.158	-0.055	-1.639	-0.259	0.158	-0.055	-1.641					
**3. Demand T1 × Sleep T1**													*R*^2^ = 0.064; Δ*R*^2^ = 0.002				
Demand **×** Flexibility									-0.086	0.135	-0.020	-0.635	*F*_14,1174_ = 5.77, *p* < 0.01;				
Demand **×** Languidity									-0.243	0.141	-0.056	-1.726	Δ*F*_3,1174_ = 1.03, *p* > 0.38				
Demand **×** Morningness									-0.171	0.154	-0.038	-1.110					

*T1, first time data (2008/2009); T4, fourth time data (2012); *B*, unstandardized regression coefficient; SEB, unstandardized standard error; β, standardized regression coefficient; *t, t*-test; p, probability; *R*^*2*^, squared multiple correlation coefficient; Δ*R*^*2*^, change in *R*^*2*^ between steps; *F, F*-value with corresponding degrees of freedom; Δ*F*, change in *F* between steps. ^*1*^Living without children at both T1 and T4 comprises the reference category, ^∗^*p* < 0.05, ^∗∗^*p* < 0.01.*

After entry of all independent variables at Step 3, the variance explained by the model as a whole was 6.4%, *F*_14,1174_ = 5.77, *p* < 0.01; *F*^2^ = 0.068 (small-medium effect). In the final model, T1 demands (β = 0.167, *p* < 0.01; semi-partial correlation = 0.160; small-medium effect), T1 flexibility (β = 0.084, *p* < 0.01; semi-partial correlation = 0.079; small effect), and T1 social support (β = -0.070, *p* < 0.05; semi-partial correlation = -0.066; small effect) were significantly associated with workaholism at T4. The results from the final model further showed that demographic control variables in terms of not living with children at T1 but T4 (β = -0.102, *p* < 0.01; semi-partial correlation = -0.095; small effect) and age (β = -0.066, *p* < 0.05; semi-partial correlation = -0.058; small effect) also contributed significantly.

## Discussion

To the best of our knowledge, this study is the first to examine links between workaholism and sleep, including chronotype (morningness), flexibility, and languidity. Both workaholism (Clark et al., 2016) and sleep (Irwin, 2015) are known predictors of health, and understanding their possible inter-relationships is informative for constructing conceptual models of long-term pathways to health. Our findings show that among the work variables, job demands and social support are the most important correlates of workaholism; while flexibility seems to be the most important sleep variable associated with workaholism. The findings are discussed in further detail below.

### Working Conditions and Workaholism

#### Job Demands

Job demands were positively associated with workaholism. This finding supports our first hypothesis and previous studies (Johnstone and Johnston, 2005; Choi, 2013; Matsudaira et al., 2013; Shimazu et al., 2014). Demands may act as a stressor, and job stress may feel uncomfortable, making the person work harder in an attempt to escape (negative reinforcement). It is also conceivable that chronic high demands act as cues signaling what the norms are in an organization – to work excessively hard. Thus, demands may serve as discriminants and motivational cues in the workaholic behavior chain.

#### Job Control

We found no support for a negative relationship between job control and workaholism (H2: rejected), which was based on the assumption that high job control does not necessitate overwork as a measure to counteract an uncontrollable working situation. Still the result is in line with two previous studies (Guglielmi et al., 2012; Shimazu et al., 2014). The low reliability of the job control scale in our study may also explain the lack of association with workaholism.

#### Social Support

Social support at work was negatively related to workaholism (H3: supported). Although previous studies have shown inconsistent findings regarding the relationship between the two constructs (Choi, 2013; Matsudaira et al., 2013; Shimazu et al., 2014), the hypothesis was supported. It has been suggested that a socially supportive climate may prevent workaholism from developing in the first place or diminish the negative consequences of it through helping employees to enjoy their work (Johnstone and Johnston, 2005). Other mechanisms may also be involved, such as social distraction from work and instrumental help so that one does not need to do everything alone. An additional speculation is that some may work obsessively in an environment where social support is lacking, in order to gain support and social recognition. Finally, being addicted to work may also imply that there is little time for interaction with colleagues, thus undermining the potentially positive impact of social support.

### Sleep Variables and Workaholism

#### Sleep Flexibility

We also found support for a positive association between the ability to sleep and work at odd times (i.e., flexibility) and workaholism (H4: supported). Although flexibility

(Di Milia et al., 2005) is mainly regarded as a desirable attribute enabling good adaptation to shift work, it was still expected to be positively related to workaholism as the former constitutes a predisposition, which would make it possible to work beyond what is normally expected. The finding may be especially relevant for shift workers, where high score on sleep flexibility would enable them to take on extra work occurring at different times (i.e., shifts). The finding is as such in line with studies showing that flexibility is positively related to shift work tolerance (Saksvik et al., 2011). A recent study among police officers showed that those reporting high ability to work and sleep at odd times also preferred more consecutive night shifts than those with lower scores (Nabe-Nielsen et al., 2016), which again suggests that flexibility is associated with heavy work investment.

#### Sleep Languidity

By contrast, vulnerability-to-sleep loss like being slack, slow, and sleepy after cutting down on sleep (i.e., languidity) was unrelated to workaholism (H5: rejected). This was unexpected as previous studies have shown languidity to be related to worsening of insomnia symptoms over time (Vedaa et al., 2016) and has been shown to be negatively associated with sleep-related shift work tolerance for the day, evening, and night shift, respectively (Storemark et al., 2013). The hypothesis concerning the inverse relationship between languidity and workaholism was based on the assumption that people with difficulties overcoming drowsiness and feelings of lethargy following a reduction in sleep would find it challenging to work hard and for extended periods. Noteworthy, as workaholism is characterized by overwork and sometimes exhaustion, then languidity could be a possible consequence (of chronic fatigue) rather than a “predictor” of workaholism. Future studies should thus address the directionality between languidity and workaholism, preferably based on cross-lagged associations.

#### Morningness

Furthermore, we found no support for a relationship between morningness and workaholism (H6: rejected). This hypothesis was based on findings showing that morningness in the majority of previous studies is positively associated with shift work tolerance and studies showing that morningness is inversely related to chronic work-related fatigue (Martin et al., 2012). It should be noted that a negative and significant zero-order correlation coefficient between morningness and workaholism was found in the present study.

The non-significant effect of morningness when being regressed on workaholism may thus reflect redundancy between morningness and other predictors in the model.

A more substantially based explanation for the non-significant finding, when controlling for the other predictors, may be that individuals with high scores on morningness (“morning larks”) cope well with morning work and have a high working capacity in the morning, but cope more poorly and have lower working capacity in the evening/night, whereas the opposite pattern is expected for those with low scores on morningness (“night owls”). The overall working capacity across all times of the day may therefore be independent of the scores on the morningness dimension, especially for a shift-working population such as the present one.

### Job Demands and Sleep Interaction Effects

Finally, we expected that demands would interact with the sleep/wake patterns in explaining workaholism, but none of the interaction effects turned out to be significant (H7: rejected). Thus, demands do not seem to predict workaholism over time depending on the levels of (a) flexibility, (b) languidity, or (c) morningness. This is in line with a recent study conjointly investigating dispositional (big five) and organizational characteristics as possible predictors of workaholism, in which none of the interaction terms had a significant effect on workaholism (Schaufeli, 2016). These findings reflect that (rather than being dependent) situational and dispositional antecedents of workaholism appear to have independent impact. Still, as some other studies have found evidence for an interaction between person characteristics and work culture (Mazzetti et al., 2014) in predicting workaholism, the idea that work-related and individual differences may interact and have a combined influence on workaholism should be investigated in future research, including other samples as well as a wider array of work-related and individual differences.

### Practical Implications

Despite the fact that several of the predictors included in the analysis reached statistical significance, the strengths of the associations were relatively weak. In terms of the semi-partial correlation coefficients, work demand was the strongest predictor. This may suggest that workaholism may be prevented by decreasing workload and external pressure (Mazzetti et al., 2014), by, for example, changing expectations and norms. Social support was inversely related to workaholism and suggests that interventions aiming to increase social support may have positive effects in this realm as well, in addition to positive effects of such interventions that have been previously demonstrated for several health-related outcomes (Wagner et al., 2015). Interventions that might improve demands and social support entail increased task variety, more personnel, more time to plan work, and more teamwork (Bambra et al., 2007). In terms of the sleep variables, it seems that subjects with high levels of flexibility may run a higher risk than others in terms of developing workaholism over time. In such cases, career counseling may be a useful intervention (Kirk and Brown, 2003). Relevant counseling strategies may include stress management and helping workaholics to find work they enjoy or work that they perceive as highly meaningful (Bonebright et al., 2000). Furthermore, it has been recommended that a specific counseling goal for compulsive workaholics might be to reduce the extent to which their behavior is perceived as dysfunctional by themselves and by the organization employing them (Naugthon, 1987). Counseling based on self-validation has also been suggested, where the workaholic learns to validate and value other self-related aspect than work, such as the spiritual, transcultural-existential, social-cultural, and familial and physical self (Ishiyama and Kitayama, 1994).

### Strengths and Limitations

Variance explained by the independent variables was relatively small, which should spur future studies to identify factors able to explain larger proportions of the variance in workaholism. Certain variables assessed at T1 were associated with workaholism at T4, but this does not demonstrate directionality or causal relationships. The sample was quite homogenous (profession) which limits the range of confounders influencing the results; but this also puts restrictions on the generalizability to other populations. The low response rate at baseline may have influenced the cohort, and we cannot rule out that the survey topic influenced participation. Lack of knowledge about non-responders, however, prevents us from drawing firm conclusions about this. However, compared to a Swedish representative sample of workers, the nurses in our study did not deviate much on the mean scores on demand, control, and support (Chungkham et al., 2013), suggesting that the sample on some central variables may be representative. Further, the job control scale had very low internal consistency (α = 0.49) that typically weakens relationships with other constructs. Another, perhaps most profound, limitation involves not controlling for level of workaholism at T1 (assessed at T4 only). Work variables (demands, control, and support) measured at T1 may also have changed over the 2-3-year period, in addition to the individual differences (flexibility, languidity, and morningness) – although generally regarded as stable. As we did not measure these at T4, we cannot be sure whether what took place 2–3 years ago matters now. This complicates the interpretation and may explain why the amount of variance explained by the variables in our study was low.

In terms of strengths, the present study had a large sample size providing high statistical power. Independent variables were assessed 2–3 years before the dependent variable. Investigating the reverse “directionality” would, however, have been more ideal (e.g., in autoregressive models), but data for workaholism is so far only available from T4, and several of the independent variables have only been assessed at T1. Making interferences about directionality with the current design is therefore impossible. This study still represents an improvement compared to most other studies in this area that mainly rely exclusively on cross-sectional designs. The fact that the independent and the dependent variable(s) were assessed at different time points reduces the risk of the results being distorted by the common method bias (i.e., self-report) (Podsakoff et al., 2003). Another asset is the use of valid instruments embedded within solid theoretical frameworks. Finally, we investigated constructs (flexibility, languidity, and morningness) that never have been related to workaholism previously. Thus, the present study expands our conceptual understanding of workaholism.

## Conclusion

The present study is the first to examine associations between working conditions, dispositional sleep variables, and workaholism. The results indicate that job demands (positively), social support at work (negatively), and sleep flexibility (positively) are associated with workaholism over a 2-3-year time period. We summarize the study results in the context of a conceptual model that bridges theories of wokaholism development with working conditions and dipositional sleep factors. Altough several questions remain, this framework makes specific, testable predictions that can be examined in future studies. Future research should investigate the relationship between these variables with longitudinal designs, preferably with all variables assessed in all waves, in order to reveal the directionality of the relationships. A further examination of the long-term associations between these factors might help prevent the development of workaholism by intervening on specific dysfunctional working conditions and providing counseling regarding sleep variables associated with workaholism.

## Ethics Statement

The study procedures were carried out in accordance with the Declaration of Helsinki and the Norwegian Health Research Act. The study was approved by the Regional Committee for Medical and Health Research Ethics (REC West) (no. 088.88) and the Norwegian Data Inspectorate (08/01235/IUR). All participants signed an informed consent form before being included in the study.

## Author Contributions

SP, BB, BM, and NM provided substantial contributions to the conception or design of the work and the acquisition of the data. CA and SP conducted the analysis, whereas AB, AS, and JH contributed to the interpretation of data for the work. CA and SP drafted the work. AB, BB, BM, NM, AS, and JH revised the work critically for important intellectual content. All authors approved the final version to be published and agreed to be accountable for all aspects of the work in ensuring that questions related to the accuracy or integrity of any part of the work are appropriately investigated and resolved.

## Conflict of Interest Statement

The authors declare that the research was conducted in the absence of any commercial or financial relationships that could be construed as a potential conflict of interest.
